# Induction of interferon response by high viral loads at early stage infection may protect against severe outcomes in COVID-19 patients

**DOI:** 10.1038/s41598-021-95197-y

**Published:** 2021-08-03

**Authors:** Eric C. Rouchka, Julia H. Chariker, Brian Alejandro, Robert S. Adcock, Richa Singhal, Julio Ramirez, Kenneth E. Palmer, Amanda B. Lasnik, Ruth Carrico, Forest W. Arnold, Stephen Furmanek, Mei Zhang, Leslie A. Wolf, Sabine Waigel, Wolfgang Zacharias, Jose Bordon, Donghoon Chung

**Affiliations:** 1 Department of Computer Science and Engineering, University of Louisville, Louisville, KY USA; 2 Kentucky IDeA Network of Biomedical Research Excellence (KY-INBRE) Bioinformatics Core, Louisville, KY USA; 3 Department of Neuroscience Training, University of Louisville, Louisville, KY USA; 4 Department of Microbiology and Immunology, School of Medicine, University of Louisville, Louisville, KY USA; 5 Center for Predictive Medicine, University of Louisville School of Medicine, Louisville, KY USA; 6 Department of Medicine, University of Louisville, Louisville, KY USA; 7 Division of Infectious Diseases, University of Louisville, Louisville, KY USA; 8 Department of Pharmacology and Toxicology, University of Louisville, Louisville, KY USA; 9 James Graham Brown Cancer Center, University of Louisville, Louisville, KY USA; 10 Genomics Core Facility, University of Louisville, Louisville, KY USA; 11 Washington Health Institute, George Washington University School of Medicine, Washington, D.C USA; 12 Department of Medicine, George Washington University School of Medicine, Washington, D.C USA

**Keywords:** SARS-CoV-2, Infection

## Abstract

Key elements for viral pathogenesis include viral strains, viral load, co-infection, and host responses. Several studies analyzing these factors in the function of disease severity of have been published; however, no studies have shown how all of these factors interplay within a defined cohort. To address this important question, we sought to understand how these four key components interplay in a cohort of COVID-19 patients. We determined the viral loads and gene expression using high throughput sequencing and various virological methods. We found that viral loads in the upper respiratory tract in COVID-19 patients at an early phase of infection vary widely. While the majority of nasopharyngeal (NP) samples have a viral load lower than the limit of detection of infectious viruses, there are samples with an extraordinary amount of SARS-CoV-2 RNA and a high viral titer. No specific viral factors were identified that are associated with high viral loads. Host gene expression analysis showed that viral loads were strongly correlated with cellular antiviral responses. Interestingly, however, COVID-19 patients who experience mild symptoms have a higher viral load than those with severe complications, indicating that naso-pharyngeal viral load may not be a key factor of the clinical outcomes of COVID-19. The metagenomics analysis revealed that the microflora in the upper respiratory tract of COVID-19 patients with high viral loads were dominated by SARS-CoV-2, with a high degree of dysbiosis. Finally, we found a strong inverse correlation between upregulation of interferon responses and disease severity. Overall our study suggests that a high viral load in the upper respiratory tract may not be a critical factor for severe symptoms; rather, dampened antiviral responses may be a critical factor for a severe outcome from the infection.

## Introduction

The severe acute respiratory syndrome coronavirus 2 (SARS-CoV-2) causing the coronavirus disease of 2019 (COVID-19) has resulted in a death toll of more than 1.9 millions of human lives worldwide as of January 8th 2021 since its outbreak in December 2019 and the impacts continue^1^. These massive burdens are mainly due to its high transmissibility and severe pathogenicity. Studies have found that SARS-CoV-2 has an R_0_ as high as 3.6, much greater than influenza virus^2^. In addition, a significant number of people infected with SARS-CoV-2 might have not been properly diagnosed nor reported^3^, posing a challenge in preventing community-based spread of SARS-CoV-2.

The spectrum of the clinical outcomes from SARS-CoV-2 infections ranges from lack of symptoms to severe illnesses comprising the acute respiratory distress syndrome and multisystem inflammatory syndrome^4^. Aging, male sex and chronic comorbidities such as diabetes mellitus and arterial hypertension have been reported as host factors associated with the severity of illness and fatality in many cases but not in all^5^.

Outside of host comorbidities, our understanding in COVID-19 pathogenesis is still limited, largely due to the complex interactions among multiple virological, microbiological, and host factors contributing to pathogenic outcomes of COVID-19. Understanding of the viral dynamics of SARS-CoV-2 and host responses driving the pathogenic mechanisms in COVID-19 are evolving rapidly. Innate immune responses including type I interferons are known to be important to control the replication of SARS-CoV-2^6–9^. The viral load has been reported as an important factor in efficient induction of host responses, including type 1 interferon responses^10^. However, at the same time, high viral load has been suggested to contribute to mortality. A clinical study found an independent association between viral load and mortality with a 7% increase in hazard for each log transformed viral RNA copy number^11^. In addition, Westblade et al. reported that the hospital admission viral load independently predicts mortality in COVID-19 patients with and without cancer^12^. On the contrary, Kimon et al. presented an inverse correlation between viral loads in NP swabs and clinical outcomes and duration of symptoms^13^.

Importantly, no studies have shown all of these factors within a defined cohort to understand their interactions. To address this important question, we sought to understand how four key viral pathological components; (1) viral load, (2) viral strain, (3) host response to the infection and (4) co-infection, interplay with each other in a COVID-19 patient cohort and how they contribute to clinical outcomes.

Our study of patients with SARS-CoV-2 aimed (1) to examine the continuum of viral load in nasopharyngeal (NP) swabs, (2) to determine the meaning of this variety in viral load in relation to the virus dynamics and host responses and, (3) to understand if different viral loads can be related to pathogenic factors for the spectrum of the COVID-19 clinical outcomes.

We found a strong correlation between interferon responses and viral load, which inversely correlates with clinical severity. We postulate an interplay of these three factors where a strong up-regulation of antiviral responses mediated by high viral loads at the initial phase have led to protective immune responses.

## Results

### Sample collection

**Figure 1:**
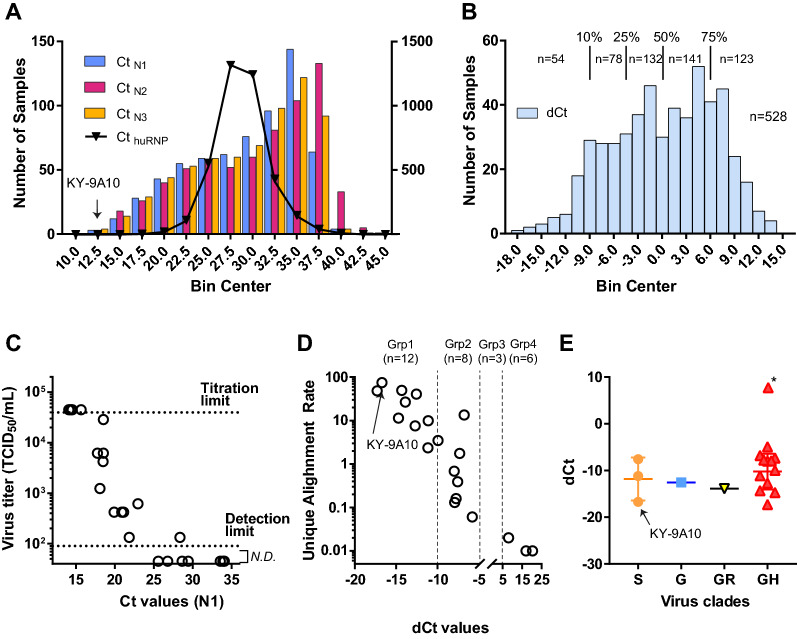
Virological characterization of NP samples based on their viral RNA loads. (**A**) Histogram analysis of a total of 3,640 SARS-CoV-2 test samples based on the Ct values of N1,N2,N3 and huRNP. A total of 544 samples were determined to be positive for SARS-CoV-2 (**B**) Histogram analysis of a total of 528 SARS-CoV-2 positive samples with a valid dCt value based on dCt values. (**C**) Comparison between virus titers (TCID50/mL, y axis) and viral RNA load (Ct_N1_) in nasopharyngeal swabs. Viral titers were determined in a TCID50 formal with 12-well plates. (**D**) Proportion of viral sequences in the total RNA in the function of dCt. (**E**) Virus clades detected in the samples and their viral RNA load in the samples. **S**: C8782T, T28144C includes NS8-L84S. **L**: C241,C3037,A23403,C8782,G11083,G25563,G26144,T28144,G28882 (WIV04-reference sequence). **V**: G11083T,G26144T NSP6-L37F + NS3-G251V. **G**: C241T,C3037T,A23403G includes S-D614G. **GH**: C241T,C3037T,A23403G,G25563T includes S-D614G + NS3-Q57H. **GR**: C241T,C3037T,A23403G,G28882A includes S-D614G + N-G204R.

NP swab samples collected from in- and out-patients within the area of Louisville (KY, USA) were tested for SARS-CoV-2 with the real-time PCR assay developed by the US CDC^14^ (See “Methods and Materials” for details). To understand the distribution of viral RNA loads in clinical NP samples, we first analyzed a cohort of 3,640 samples that were collected between 3/11/2020 and 4/11/2020. A total of 544 samples were determined as positive (Ct values of less than 39 for both N1 and N2) for SARS-CoV-2. The overall positive rate was 14.95% and the median Ct values for viral targets (N1, N2, and N3 probe/primer pairs for the viral Np gene) were between 30.84, 31.86, and 30.98. The median Ct of the Human RNase P (Hu RNaseP, endogenous control) target was 28.63 (Table 1, Fig. 1A). The lowest Ct value for viral targets was 11.58 (N2 target) from a sample (sample ID: KY-9A10) with Ct values of 13.3, 12.43, and 33.9 for N1, N3, and Hu RNaseP target, respectively.

**Table 1:** Distribution of SARS-CoV-2 viral RNA loads in patients.

	Ct _N1_	Ct _N2_	Ct _N3_	Ct _huRNP_	dCt
Number of values	663	674	661	3840	555
Minimum	13.3	11.58	12.43	16.26	−17.56
5% percentile	17.98	17.34	17.27	24.25	−10.56
10% percentile	20.02	19.85	19.69	25.39	−8.81
25% percentile	24.58	24.88	24.3	26.91	−4.70
Median	30.84	31.86	30.98	28.63	0.97
75% percentile	34.74	36.33	35.01	30.36	5.79
90% percentile	36.38	37.98	36.57	32.22	9.00
95% percentile	37.08	38.96	37.26	33.78	11.31
Maximum	44.63	42.13	42.48	41.78	36.54
Mean	29.36	30.33	29.39	28.72	1.03
Std. deviation	6.261	6.977	6.493	2.872	8.27
Std. error of mean	0.243	0.2687	0.2526	0.0463	0.35
Lower 95% CI of mean	28.88	29.8	28.9	28.63	0.34
Upper 95% CI of mean	29.84	30.86	29.89	28.81	1.72

A histogram analysis of the Ct values showed that the distribution of viral RNA load is not symmetric around the median; rather, it skewed negatively. Unlike the viral RNA, the distribution of Ct values for Hu RNAseP was close to a normal symmetric distribution, supporting appropriate sample collection for the study (Fig. 1A). This analysis showed that viral RNA load in the upper respiratory tracts varies significantly and while more than 50% samples showed a lower viral RNA load with Cts > 31, some patients (e.g., KY-9A10) carried a high viral RNA load, ~ 2^20^-fold higher than the median (2 ^Ct median – Ct sample^) .

### Analysis of stratified samples

To understand critical confounding factors determining viral loads in the upper respiratory tracts, we sought to characterize the nasopharyngeal samples in detail. Samples were categorized into five groups based on the dCt values, defined as the difference between the Ct value of the reference gene (huRNP, Ct_huRNP_) and the average Ct values of N1, N2, and N3 targets (Ct_ave_): (1) Group 1 for top 10% (− 10.56 < dCt < − 8.8); Group 2 for top 10–25% (− 8.8 < dCt < − 4.7); Group 3 for top 25–50% (− 4.7 < dCt < 0.97); Group 4 for the bottom 50% (dCt > 0.97) ; and negative for any of SARS-CoV-2 viral target (Table 1). A total of 3–6 samples were randomly selected from each dCt group except the top 10% group where 12 samples were chosen. A total of 34 samples were selected for full characterization (Table 2).

**Table 2:** Characterization of 34 samples used in the study.

Group	Sample ID	Ct average	dCt	Sex	Age	Severity	Virus Clade
Group 1 (Top 10%)	KY-50G10	13.38	− 17.29	Female	91	Mild	GH
	KY-9A10	12.44	− 16.71	Male	74	Moderate *#	S
	KY-9A06	17.28	− 14.73	Male	34	Mild	GH
	KY-28D08	13.98	− 14.33	Male	56	Mild	GH
	KY-28F11	14.96	− 13.9	Female	39	Mild	GR
	KY-52D08	13.96	− 12.7	Female	30	Mild	GH
	KY-63G09	14.15	− 12.55	Female	82	Mild	G
	KY-53C10‡	20.75	− 12.53	Male	54	Mild	NT
	KY-29G02‡	15.48	− 11.71	Female	90	Severe *	NT
	KY-51C11	17.72	− 11.17	Male	27	Mild	S
	KY-03G06	18.04	− 11.12	Female	88	Mild	GH
	KY-39C07	18.34	− 9.94	Female	85	Mild	GH
Group 2 (Top 10–25%	KY-37H09‡	18.67	− 7.99	Female	89	Mild	GH
	KY-48H07‡	19.8	− 7.92	Male	52	Moderate	GH
	KY-52F02	21.58	− 7.75	Female	45	Moderate	GH
	KY-9E09	25.54	− 7.57	Male	66	Moderate	S
	KY-48E10	19.97	− 7.33	Female	21	Mild	GH
	KY-48D04	20.96	− 6.77	Female	29	Mild	GH
	KY-50F07‡	25.49	− 5.8	Female	94	Mild	ND
	KY-51C03‡	23.33	− 4.93	Female	85	Moderate	GH
Group 3 (Top 25- 50%	KY-52D06	27.74	− 0.57	Male	55	Mild	ND
	KY-53B02	29.35	− 0.44	Female	40	Mild	ND
	KY-51H11	26.93	− 0.09	Female	62	Severe*	ND
Group 4 (Bottom 50%)	KY-37F04	29.89	1.3	Male	59	Severe	ND
	KY-39C10	28.76	1.67	Female	93	Mild	ND
	KY-37A05	34.52	8.03	Female	88	Severe	GH
	KY-51H03	36.27	8.3	Male	60	Severe	ND
	KY-37D02	35.39	10	Male	40	Moderate	ND
	KY-39H02‡	34.65	10.57	Male	75	Severe	ND
Group 5 (Negative)	KY-37B01	> 40	> 12.02	Female	69	unknown	ND
	KY-37A09	> 40	> 13	Female	93	unknown	ND
	KY-37A01	> 40	> 13.99	Male	44	unknown	ND
	KY-37A07	> 40	> 14.99	Male	82	unknown	ND
	KY-37A03	> 40	> 15.4	Female	49	unknown	ND

*Patients with a lethal outcome.

^#^COVID-19 patient died, but this patient had DNR/DNI advanced directives. The patient did refuse to be intubated.

^‡^Samples were unavailable or removed from the host gene expression analyses due to the quality of the NGS readings.

To confirm the viral RNA loads in the selected samples, 24 specimens out of the 34 samples were re-tested independently in two secondary SARS-CoV-2 coronavirus assays using the Luminex ARIES® platform: 1) ARIES® SARS CoV 2 Assay (EUA) targeting the ORF1ab and N, and 2) ARIES® SARS-CoV-2 LDT Assay targeting N1 and N3. These three assays demonstrated consistent results (Supplementary Table 1) and correlation analysis of Ct values between all comparison groups were > 0.9 except one for the IVD_orf and CDC N1 target (r = 0.893). Test performance for individual samples are listed in Supplementary Table 2 (Supplementary Table 2). This analysis showed these three viral RNA load quantitation methods have a similar sensitivity and produced consistent results.

### In vitro virological characterization

We then examined if samples with a lower Ct value in fact had a high infectious viral load. To test this, we directly titrated the viral load in the NP swab samples by using an in vitro cell culture TCID_50_ assay. Sterile filtered samples were added to cultures of Vero E6 cells grown in 12-well plates with four replicates for each dilution. A total of 28 samples including five of the Group 6 samples (negative for SARS-CoV-2) were tested (Fig. 1C). We found that samples with Ct < 16 for CDC N1 showed a virus titer > 45,000 TCID_50_/mL and samples with Ct > 25 did not show any virus replication in the cells except one sample (Ct = 28.36 and 133 TCID_50_/mL). Samples with Ct between 16 and 25 showed a reverse correlation between Ct values and infectious virus titer, indicating samples with a low Ct value indeed contained a high viral load. This was further confirmed by comparison between the proportion of viral sequences detected by the next-generation sequencing (see below) and their Ct values (Fig. 1D).

### Genetic characterization of viral genomes found in high viral load samples

Our analysis clearly demonstrated that some patients had a significantly high viral load than others. To understand if the difference in viral load in the NP swabs is due to viral factors, we sought to determine and compare the viral genome sequences from the samples. A total of 32 samples were subjected to high-throughput sequencing. The total RNA was isolated and sequenced on the Illumina NextSeq 500 platform (See “Methods and Materials” for the sequence analysis and alignment). The total sequence reads are available from the NCBI’s SRA (bioproject PRJNA626685). Among the 32 samples, six samples failed quality control checks and the remaining 29 samples showed data with a quality warranting a further analysis.

From the NGS data, we were able to obtain the full viral sequences from 18 samples (Table 2). To understand if a specific viral clade can generate a higher viral load than the others, we categorized the isolates based on viral clades and dCt. Currently there are six different clades of circulating SARS-CoV-2 reported^15^ and some clades including G and GH are known to produce a higher virus titer both in vitro and in patients^16,17^. In our samples, we found four different viral clades in our samples: S, G, GR, and GH. The dominant GH clade was detected in 13 samples, followed by S, G, and S clade detected in 3, 1, and 1 samples, respectively (Fig. 1D). This pattern is consistent with research reported by others^15^, showing a high prevalence of the GH type in the North America. When we compared the dCt values of the samples based on the virus clade, all clades showed an average dCt between -12.55 (G clade) and -10.2 (GH clade, except an outlier, KY-37A05, with a dCt of 8.03), indicating a similar viral load among the clades.

### Clinical correlates

**Figure 2:**
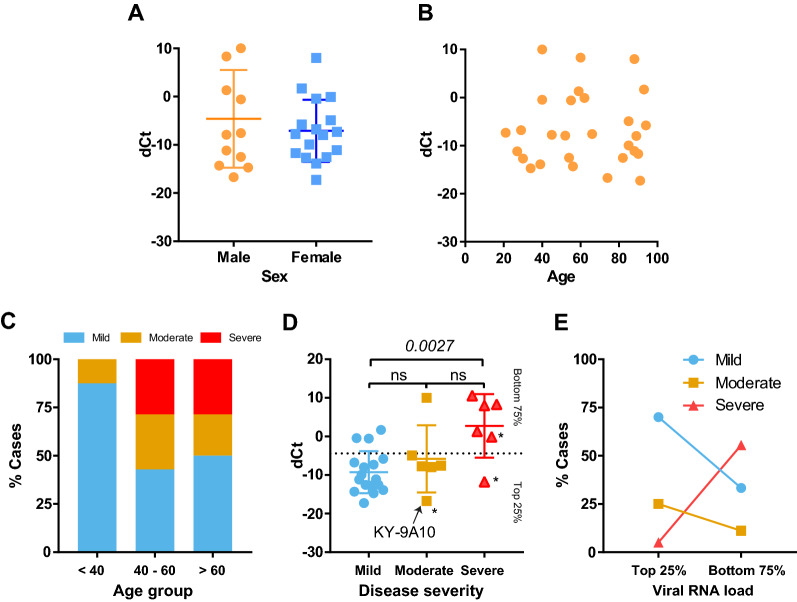
Relationship between dCt and clinical data for selected samples. (**A**) dCt distribution of between male and female. (**B**) dCt distribution based on patient age. (**C**) Disease severity based on age. (**D**) Analysis of dCt based on disease severity group. *Patients with a lethal outcome. (**E**) Disease severity analysis based Viral RNA load (dCt).

Next, we sought to understand if viral loads in upper respiratory tract is affected by host factors or, further, the disease severity is determined by the viral loads in the early phase of symptomatic infection. To analyze this, we profiled the viral loads in the NP samples (dCt) based on various clinical relates including; sex, age, and clinical outcomes (Fig. 2).

Neither sex (Fig. 2a) nor age (Fig. 2b) showed significant correlation with the dCt. However, we found a strong inverse correlation between the viral load and the COVID-19 disease severity of the patients. Based on the clinical assessments, we classified the COVID severity into three groups: (1) Mild (outpatients, screening samples from long-term care facilities, or an emergency department visit with no hospital admission); (2) Moderate (hospital admission to ward), and (3) Severe (hospital admission to an intensive care unit within first 48 h). According to this grouping, a clear age-disease severity relationship was shown (Fig. 2c), validating our grouping strategy. The Mild group showed the overall highest viral load with an average dCt of − 9.26, and the Severe group showed the least (dCt ave = − 2.73). The difference of dCt between the groups were significant (*P* = *0.0027*, Tukey's multiple comparisons test). There were three cases with a lethal outcome: two cases in the Severe group and one in the Moderate group (KY-9A10). The one patient (74 years old) in the Moderate group had Do-Not-Resuscitate (DNR) and Do-Not-Intubate (DNI) advanced directives. Despite the overall negative correlation between the viral load and disease severity, two cases with a lethal outcome showed a higher viral load in their NP samples, which may indicate a possibility that a high viral load may lead a critical clinical outcome in some patients.

### Host response

Our analysis of the viral variants showed that some COVID-19 patients have high amounts of viral load in the upper respiratory tract, and the viral clades may not be a critical determinant of high viral loads in early symptomatic infections.

**Figure 3:**
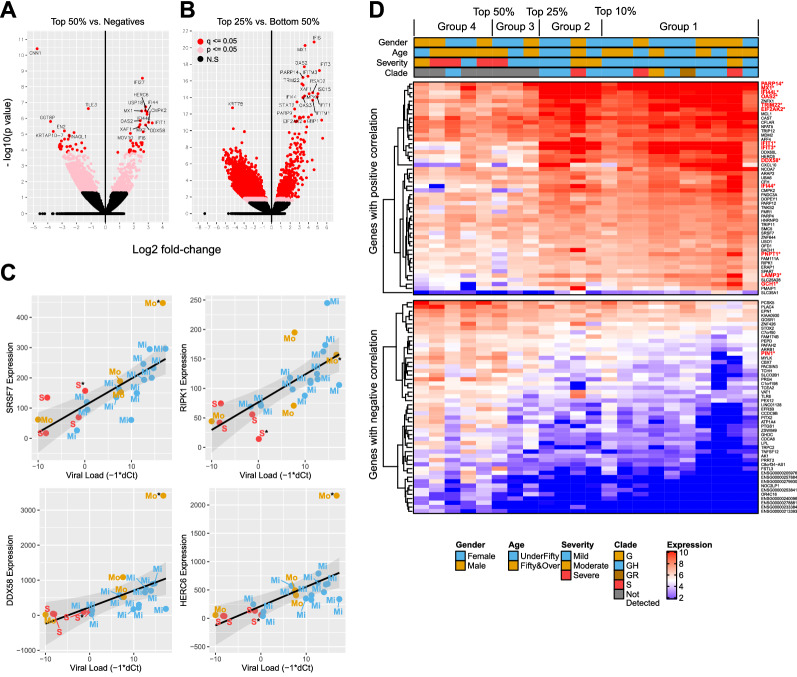
Comparative analyses of host gene expression profile in SARS-CoV-2 patients based on viral load. (**A**) Volcano plot analysis identified that interferon related genes are up-regulated in SARS-CoV-2 infected group compared to the Negative control. (**B**) The Top 25% group showed a higher level of expression of interferon related genes compared to the Bottom 50% group. (**C**,**D**) Representative genes identified with our correlation analysis between viral loads and individual gene expression level. S, Mo, and Mi denote the Severe, Moderate, and Mild groups. *Fatal cases (**C**). Gene expression across samples displayed in order of increasing viral load for positive (top) and negative (bottom) correlations (**D**).

To see what host responses are specific to the high viral load samples, we profiled the host gene expressions using RNA-sequencing approaches. We first investigated what genes are expressed in the NP upon infection. We compared the Top 50% (Group 1, 2, and 3) against the negative control and found that a total of 60 genes were differentially expressed (35 upregulated and 25 downregulated genes in the Top 50% group, Fig. 3a). Many interferon stimulated genes (e.g., interferon alpha inducible (IFI) genes, HERC6, OAS2, and DDX58) were significantly up-regulated in the infected group compared to the negative group (Table 3 and Supplementary Table 3 for down-regulated genes).

**Table 3:** Top 20 up-regulated genes in the Top50 group compared to the Negative control group.

Ensembl ID	Gene Symbol	Description	Log_2_FC	P-value	Q-value
ENSG00000165949	IFI27	Interferon alpha inducible protein 27	2.594	2.862e−09	3.337e−05
ENSG00000138642	HERC6	HECT and RLD domain containing E3 ubiquitin protein ligase family member 6	2.823	1.857e−07	0.001
ENSG00000184979	USP18	Ubiquitin specific peptidase 18	2.811	3.238e−07	0.001
ENSG00000157601	MX1	MX dynamin like GTPase 1	2.541	3.411e−07	0.001
ENSG00000137965	IFI44	Interferon induced protein 44	2.919	3.959e−07	0.001
ENSG00000137959	IFI44L	Interferon induced protein 44 like	2.921	5.501e−07	0.001
ENSG00000111335	OAS2	2'-5'-Oligoadenylate synthetase 2	2.438	1.394e−06	0.003
ENSG00000134326	CMPK2	Cytidine/uridine monophosphate kinase 2	3.027	1.771e−06	0.003
ENSG00000185745	IFIT1	Interferon induced protein with tetratricopeptide repeats 1	3.286	1.936e−06	0.003
ENSG00000183486	MX2	MX dynamin like GTPase 2	2.427	2.470e−06	0.004
ENSG00000107201	DDX58	DExD/H-box helicase 58	2.818	2.615e−06	0.004
ENSG00000132530	XAF1	XIAP associated factor 1	2.349	3.466e−06	0.005
ENSG00000126709	IFI6	Interferon alpha inducible protein 6	2.647	7.057e−06	0.009
ENSG00000155363	MOV10	Mov10 RISC complex RNA helicase	1.750	8.844e−06	0.010
ENSG00000073605	GSDMB	Gasdermin B	1.765	2.569e−05	0.023
ENSG00000078081	LAMP3	Lysosomal associated membrane protein 3	2.485	3.409e−05	0.027
ENSG00000137198	GMPR	Guanosine monophosphate reductase	2.580	3.427e−05	0.027
ENSG00000138496	PARP9	Poly(ADP-ribose) polymerase family member 9	1.913	3.679e−05	0.027
ENSG00000188313	PLSCR1	Phospholipid scramblase 1	2.151	3.711e−05	0.027
ENSG00000177409	SAMD9L	Sterile alpha motif domain containing 9 like	2.568	4.234e−05	0.029

To understand what host factors are related to the viral loads, we used two approaches: (1) comparison between viral load groups, and (2) correlation studies. A clear comparison was detected when the Top 25% samples (Group 1 and 2) were compared to the Bottom 50% group (Fig. 3a,b). We found that the Top 25% samples showed a significantly high level of expression of genes that are related with type I interferons compared to the Bottom 50% group (Table 4). Type 1 interferon signaling pathway was identified with a high significance from a gene ontology analysis for biological processes using Cluster Profiler (Supplementary Table 4).

**Table 4:** Top 20 up-regulated genes in the Top 25% group compared to the Bottom 50% group.

Ensembl ID	Gene Symbol	Description	Log_2_FC	P-value	Q-value
ENSG00000126709	IFI6	Interferon alpha inducible protein 6	4.610	2.00E−21	6.16E−17
ENSG00000157601	MX1	MX dynamin like GTPase 1	3.625	5.29E−21	8.17E−17
ENSG00000111335	OAS2	2'-5'-Oligoadenylate synthetase 2	3.543	1.95E−18	2.00E−14
ENSG00000119917	IFIT3	Interferon induced protein with tetratricopeptide repeats 3	5.193	5.61E−18	4.33E−14
ENSG00000142089	IFITM3	Interferon induced transmembrane protein 3	3.938	2.73E−17	1.69E−13
ENSG00000173193	PARP14	Poly(ADP-ribose) polymerase family member 14	3.398	3.85E−17	1.98E−13
ENSG00000132274	TRIM22	Tripartite motif containing 22	3.280	2.27E−16	1.00E−12
ENSG00000132530	XAF1	XIAP associated factor 1	3.421	3.18E−16	1.23E−12
ENSG00000134321	RSAD2	Radical S-adenosyl methionine domain containing 2	4.459	2.62E−15	9.00E−12
ENSG00000187608	ISG15	ISG15 ubiquitin-like modifier	5.034	3.49E−15	9.94E−12
ENSG00000134326	CMPK2	Cytidine/uridine monophosphate kinase 2	4.073	3.54E−15	9.94E−12
ENSG00000137959	IFI44L	Interferon induced protein 44 like	3.573	6.78E−15	1.74E−11
ENSG00000185745	IFIT1	Interferon induced protein with tetratricopeptide repeats 1	4.939	8.57E−15	2.03E−11
ENSG00000111331	OAS3	2'−5'−Oligoadenylate synthetase 3	3.396	1.04E−14	2.29E−11
ENSG00000185885	IFITM1	Interferon induced transmembrane protein 1	4.457	2.15E−14	4.42E−11
ENSG00000170581	STAT2	Signal transducer and activator of transcription 2	2.748	3.52E−14	6.79E−11
ENSG00000138496	PARP9	Poly(ADP-ribose) polymerase family member 9	2.488	2.50E−13	4.29E−10
ENSG00000117228	GBP1	Guanylate binding protein 1	4.327	1.25E−12	2.04E−09
ENSG00000055332	EIF2AK2	Eukaryotic translation initiation factor 2 alpha kinase 2	2.505	1.91E−12	2.95E−09
ENSG00000188313	PLSCR1	Phospholipid scramblase 1	3.446	2.31E−12	3.23E−09

This high confidence of the difference between viral load and interferon responses were confirmed in our second approach with a correlation analysis between viral RNA load (dCt) and individual gene expression. In this analysis, a total of 2,155 genes (484 genes with positive correlations and 1,671 with negative correlations) were identified having a significant correlation (Spearman’s Rho) at *p* < 0.01 (Tables 5 and 6). This approach identified several genes that are important for cell death (e.g., RIPK1, , and CFLAR) and RNA metabolism (SRSF7^18^, AFF4^19^, and PNPT1^20^) in addition to antiviral responses (e.g., DDX58 , IFIT1, and HERC6^21^) (Fig. 3c). A heatmap analysis with the 50 positively and negatively correlated genes (Fig. 3d) clearly demonstrated that the Top 25% group (Group 1 and 2) clearly showed a distinct gene expression pattern compared to the Bottom 75% (Group 3 and 4). Again, many of the positively correlated genes were interferon response related genes.

**Table 5:** Top 20 genes with positive correlations between viral load and expression.

Ensembl Id	HGNC Symbol	Gene Description	Estimate (r_s_)	P Value	Adj P Value
ENSG00000115875	SRSF7	Serine and arginine rich splicing factor 7	0.840	8.84E−07	0.032
ENSG00000164307	ERAP1	Endoplasmic reticulum aminopeptidase 1 Antigen processing and presentation of endogenous peptide antigen via MHC class; adaptive immune response	0.905	3.70E−06	0.045
ENSG00000137275	RIPK1	Receptor interacting serine/threonine kinase 1	0.801	9.20E−06	0.067
ENSG00000155287	SLC25A28	Solute carrier family 25 member 28	0.800	9.82E−06	0.059
ENSG00000107201	DDX58	DExD/H-box helicase 58 Positive regulation of defense response to virus; innate immune response; cytokine-mediated signaling pathway; type I interferon signaling pathway	0.796	1.19E−05	0.062
ENSG00000124201	ZNFX1	Zinc finger NFX1-type containing 1	0.793	1.42E−05	0.057
ENSG00000138035	PNPT1	Polyribonucleotide nucleotidyltransferase 1	0.788	1.79E−05	0.062
ENSG00000198887	SMC5	Structural maintenance of chromosomes 5	0.788	1.79E−05	0.062
ENSG00000003402	CFLAR	CASP8 and FADD like apoptosis regulator Regulation of apoptotic process	0.783	2.35E−05	0.071
ENSG00000047365	ARAP2	ArfGAP with RhoGAP domain, ankyrin repeat and PH domain 2	0.780	2.61E−05	0.068
ENSG00000138642	HERC6	HECT and RLD domain containing E3 ubiquitin protein ligase family member 6	0.779	2.75E−05	0.059
ENSG00000083097	DOPEY1	Dopey family member 1	0.771	3.88E−05	0.067
ENSG00000153827	TRIP12	Thyroid hormone receptor interactor 12	0.767	4.67E−05	0.068
ENSG00000185745	IFIT1	Interferon induced protein with tetratricopeptide repeats 1 Response to virus; intracellular transport of viral protein in host cell	0.765	5.11E−05	0.066
ENSG00000102699	PARP4	Poly(ADP-ribose) polymerase family member 4 Inflammatory response	0.763	5.34E−05	0.067
ENSG00000122482	ZNF644	Zinc finger protein 644	0.758	6.64E−05	0.075
ENSG00000164414	SLC35A1	Solute carrier family 35 member A1	0.745	6.78E−05	0.075
ENSG00000000971	CFH	Complement factor H Immune system process; viral process	0.757	6.92E−05	0.072
ENSG00000033178	UBA6	Ubiquitin like modifier activating enzyme 6	0.757	6.92E−05	0.072
ENSG00000072364	AFF4	AF4/FMR2 family member 4	0.754	7.53E−05	0.074

**Table 6:** Top 20 genes with negative correlations between viral load and expression.

Ensembl Id	HGNC symbol	Gene description	Estimate (r_s_)	P Value	Adj P Value
ENSG00000171703	TCEA2	Transcription elongation factor A2	− 0.816	3.54E−06	0.064
ENSG00000106992	AK1	Adenylate kinase 1	−0.806	5.77E−06	0.052
ENSG00000240096	RPL18AP1	RPL18AP1 Ribosomal Protein L18a Pseudogene 1	−0.789	1.23E−05	0.056
ENSG00000280109	PLAC4	Placenta specific 4	−0.782	2.48E−05	0.069
ENSG00000167371	PRRT2	Proline rich transmembrane protein 2	−0.771	2.62E−05	0.063
ENSG00000100307	CBX7	Chromobox 7	−0.779	2.75E−05	0.059
ENSG00000124299	PEPD	Peptidase D	−0.779	2.75E−05	0.059
ENSG00000084710	EFR3B	EFR3 homolog B	−0.769	2.86E−05	0.055
ENSG00000127445	PIN1	Peptidylprolyl cis/trans isomerase, NIMA−interacting 1	−0.773	3.70E−05	0.067
ENSG00000167925	GHDC	GH3 domain containing	−0.769	4.26E−05	0.070
ENSG00000130818	ZNF426	Zinc finger protein 426	−0.768	4.46E−05	0.071
ENSG00000134690	CDCA8	Cell division cycle associated 8	−0.756	4.56E−05	0.069
ENSG00000070404	FSTL3	Follistatin like 3	−0.755	4.72E−05	0.066
ENSG00000137491	SLCO2B1	Solute carrier organic anion transporter family member 2B1	−0.754	4.96E−05	0.067
ENSG00000213225	NOC2LP1	NOC2 like nucleolar associated transcriptional repressor pseudogene 1	−0.748	6.09E−05	0.074
ENSG00000205976	AC091951.1		−0.748	6.26E−05	0.074
ENSG00000108733	PEX12	Peroxisomal biogenesis factor 12	−0.757	6.92E−05	0.072
ENSG00000116690	PRG4	Proteoglycan 4	−0.753	7.84E−05	0.075
ENSG00000146540	C7orf50	Chromosome 7 open reading frame 50	−0.748	9.59E−05	0.083
ENSG00000165912	PACSIN3	Protein kinase C and casein kinase substrate in neurons 3	−0.744	0.000	0.089

The analyses highlighted the interferon responses as the primary response to the high viral load in the upper respiratory tract.

### Genes correlated with disease severity

Next, we sought to find host genes that correlate with disease severity. Gene expression profiles were compared between the disease severity groups; Mild, Moderate, and Severe. A differential expression analysis comparing three levels of disease severity was performed using DESeq2^22^. The number of differentially expressed genes at an adjusted *p* value cutoff < 0.05 is displayed in Table 7 for three comparisons. We sought to understand if innate antiviral responses (i.e., interferon responses) could be a determinant for mild symptoms and compared the expression of interferon-related genes between the severity groups. We found that both Mild and Moderate groups have a significantly higher expression level of interferon related genes compared to the Severe group (Table 8). In other words, the Severe group expressed interferon related genes a significantly lesser extent than the Moderate or Mild group.

**Table 7:** Summary of differentially expressed genes between the disease severity groups.

Contrast	DEGs (*q* ≤ *0.05*)
Mild vs. severe	2,368 (**210 ↑, 2,158↓**)
Mild vs. moderate	274 (**37 ↑, 237↓**)
Moderate vs. severe	2,011 (**645 ↑, 1,366↓**)

**Table 8:** Interferon related genes differentially expressed between the clinical groups.

Direction of activity	Mild vs Severe	Common to mild vs severe and moderate vs severe	Moderate vs severe
Upregulated	NFKB1, PTPN1	ADAR, BST2, DDX58, HERC5, HLA-C, HLA-E, IFI16, IFI27, IFI35, IFI6, IFIH1, IFIT1, IFIT2, IFIT3, IFITM1, IFITM2, IFITM3, IRF2*, ISG15, MX1, MX2, MYD88, NLRC5, NMI, OAS1, OAS2, OAS3, OASL, RSAD2, STAT1, STAT2, TNFAIP3, TRIM21, TRIM25, XAF1	CD14, DHX58, EP300, GBP2, HLA-A, HLA-B, HLA-F, HLA-H, IFNGR2**, IRF7*, ITCH, PSMB8, RIOK3, RIPK2, RNASEL, SHFL, TAX1BP1, TLR2, TLR3, TLR4, TLR8, TRIM38, UBE2L6, YTHDF3
Downregulated	DCST1, NLRX1, PIN1	TRAIP	POLR2F

*Interferon regulatory factor.

**Type II interferon.

### Microbial diversity

**Figure 4:**
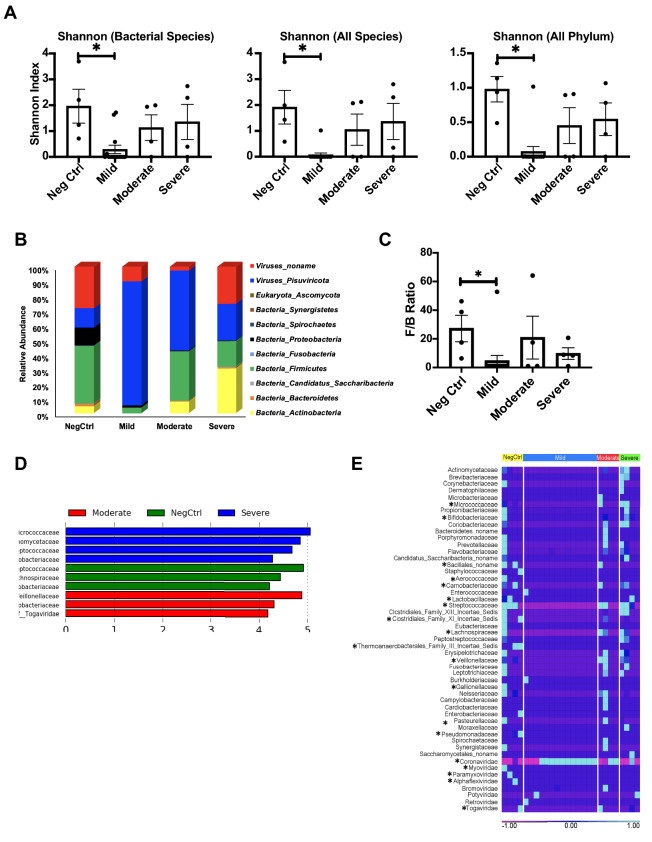
Diversity of microbiome in SARS-CoV-2 patients based on disease severity. (**A**) Metagenomic alpha diversity based on Shannon Index. (**B**) Relative abundance of phylum belonging to Kingdoms: Viruses, Eukaryota and Bacteria. (**C**) Comparison of dysbiosis (F/B ratio) based on disease severity. (**D**) Linear discriminate effect size output for microbial families belonging to all four patient groups. LDA cut-off threshold score >  = 2. No enrichment was observed in the Mild group. (**E**) Heat map representing microbial families identified in the four patient groups. Lavender signifies families that are present in low abundance or absent while light blue signifies highly abundant families. The asterisk next to the family name indicates a statistically significant difference between Mild (Highest viral load) and negative control.

The effect of SARS-CoV-2 infection on the microbial diversity within the nasopharyngeal tract was assessed and compared across the three groups. Diversity within individual samples was evaluated using an alpha diversity measure based on the Shannon Index, in order to measure species abundance and evenness across groups (Fig. 4a). Compositional changes between the samples were evaluated for their beta diversity using a JACCARD index. Our data revealed that compared to the negative control, the Mild group had a significantly reduced alpha diversity and distinct microbial composition. (Supplementary Fig. 1).

### Microbial taxonomy

Based on the significant difference in microbial diversity among the groups, we further conducted a metagenomics analysis. Taxa analysis of all kingdoms revealed that compared to all the groups, the negative control had a higher (~ 60%) bacterial microbiome. In particular, the Mild group had a significant reduction of the bacterial microbiome (contains less than 5% bacteria on average) and enhanced microbial dysbiosis marked by a decrease in F/B as compared to the negative control (Fig. 4b,c and Supplemental Table 5). Microbial family enrichment analysis of all four groups showed that the Mild group did not show any distinguishing microbial feature as compared to other groups (Fig. 4d,e). Comparatively, the negative control and the Severe group showed a healthy nasal microbiome since both show enrichment of bacterial families belonging to the Actinobacteria and Firmicutes phyla^23,24^. A heatmap for the qualitative profile of microbial families clearly demonstrates that the microbiomes of the Mild group were dominated by an expansion of Coronaviridae (p < 0.05 in comparison of negative control) (Fig. 4e). Further, statistical analysis shows there is a significant reduction of 13 bacterial and 4 viral families in the mild group as compared to the negative control (Fig. 4d and Supplemental Table 6). This decrease of microbial families not only contribute to enhanced dysbiosis but also a decrease of microbial diversity in patients with a higher viral load. Detailed statistical comparison of microbial families between groups is provided in Supplemental Table 6.

## Discussion

Viral strain, viral load, host response, and co-infection are considered as key elements of viral pathogenesis. Studies analyzing each of these factors in the function of disease severity in COVID-19 infections have previously been published by others. Kimon et al. showed an inverse relationship between viral load and disease severity^13^. A paper describing the host responses in the function of viral load was published recently, in which IFN response genes were found to be the major differentially expressed genes related to the function of viral load, a finding which our study confirms^10^. However, no studies have shown how all of these factors interplay with each other within a defined cohort. To address this important question, we investigated the interplay of these four key components in our COVID-19 patient cohort, which is comprised of patients who were admitted to the hospital with clear symptoms at a very early stage of the outbreak in the USA.

### Viral load in COVID-19 patients

Our analysis showed that COVID-19 patients have a wide range of viral loads in their upper respiratory tracts (as measured using NP swab samples), from a Ct value of 11 (KY9-A10) to Ct value of 40 (the detection limit), leading to a dynamic range > a 10E8. The sensitivity of the CDC-developed assay was equivalent to the other assay that we tested (the Luminex ARIES® platform), indicating that the detection of the high viral loads was not due to a false positive or an abnormally sensitive assay. The variance in viral load was also confirmed by direct viral titration of the NP samples. Since viral load in COVID-19 patients is known to change over the course of infection, and our study is not longitudinal, the variability that we show here could be due to the course of infection. However, all of the samples in this study were collected when the patients were admitted with symptoms; we can therefore assume that they reflect viral load within a narrow range of time after infection. Consequently, despite the limited scope of our snapshot study, it is reasonable to believe that the viral load in COVID-19 patients might vary significantly at the individual level as well. Considering that more than 75% of SARS-CoV-2–positive samples have a Ct value > 25 (Table 1), our analysis implies that the majority of SARS-CoV-2–positive patients may carry a very small load of infectious virus when they present symptoms. This could be due to higher shedding in the prodromal phase than in the symptomatic phase.

### Relation of viral load to disease severity

Viral load has been regarded as a deterministic factor for the severity of disease outcomes (i.e., more virus, worse outcomes); however, this perception has not been proven or studied in depth for COVID-19. Rather, there seems to be an inverse correlation between viral load and disease outcomes, as shown here. Kimon et al. presented a similar finding to ours, showing that viral load in NP swab samples was inversely correlated with clinical outcomes and duration of symptoms^13^. As they highlighted in the paper, these phenomena could simply be due to the difference in timing between virus replication and onset of symptoms. SARS-CoV-2 viral load reaches its peak during the pre-symptomatic stage of the disease (a median of 5.2 days post-infection), and starts to decrease after 7 days post-infection when the symptoms are fully developed^25^. Therefore, the inverse relationship between viral load and disease severity could be due to the collection of samples at different points in the progression of the disease. However, considering that all of our samples were collected at the time of admission—when patients were symptomatic—and our disease severity was determined retrospectively, we believe that the effect of collection time on viral load may be limited. Of note, we had one patient who showed a high viral load and died during the treatment (KY-9E10).

### Viral strains

The existence of such high viral load and the negatively skewed distribution of viral load in the NP samples (Fig. 1a) led us to investigate the mechanism behind the high viral load. First, we investigated whether specific viral clades or specific single nucleotide polymorphisms affected the viral load. Korber et al. showed that D614G status (G, GH, or GR clades) could be associated with high viral load compared to D614 types (e.g. S type with p = 0.037)^26^. Lorenzo-Redondo et al. also presented data^27^. However, we could not find a strong correlation between high viral load and virus type. This might be due to a relatively small number of samples in our study or a very weak correlation overall. Both papers cited, as well as Puenpa et al., reported no correlation between virus type and clinical symptoms^28^.

### Host responses

We profiled the host gene expression against the viral load with two approaches: (1) comparison between stratified groups and (2) correlation analysis between viral load and individual genes. Both analyses showed a clear correlation between up-regulation of interferon-related genes and high viral load. The difference was clearly noticeable at the 25% viral load level, or dCt < -4.7. Samples within Groups 1 and 2 showed a stastically higher expression of interferon-related genes (Fig. 3a,b). This finding was surprising to us because interferon responses are presumptively inhibitory against viral replications; however, our finding clearly showed a strong statistical association between viral load and IFN responses in the upper respiratory tract. One explanation could be that the detected viral RNA is the product of viral clearance by innate immune systems; however, we showed that the samples indeed have high virus titers as shown in Fig. 1. Rather, a better explanation of our data would be that high viral load might have induced strong IFN responses in the upper respiratory tract.

### Dysbiosis

Some reports indicated potential bacterial co-infections associated with severe COVID-19, especially *Acinetobacter baumannii*^29^. However, no information on the viral loads or interferon responses of the patients were known; hence, it is not clear if the co-infection was associated with strong viral replications or with other factors. Our metagenomics analysis indicates a relationship between dysbiosis within the nasal tract microbiota and COVID-19 severity, consistent with previous studies of the gut and respiratory microbiomes^30–37^. However, without retrospective longitudinal data on the microbiome of our patients, it is difficult to determine whether the initial infection is more likely to occur because of a pre-existing dysbiosis. This dysbiosis could also occur because of antibiotic treatments received by these patients. However, given a difference in the microbiota at different viral loads and levels of disease severity, coupled with consistency within the patient groups, the most likely conclusion is that the dysbiosis is due to SARS-CoV-2 infection, not the other way around. Our study showed no specific bacterial groups that proliferated with a high level of SARS-CoV-2 infections. Rather, we found that high viral load samples (i.e., strong Type 1 interferon responses) have almost no bacterial load in our NP samples, and NP samples with low viral loads (i.e., low in interferon responses) had similar loads of microflora to samples with no SARS-CoV-2 infection. Because our study merely shows a correlation, it is not clear if the up-regulated Type 1 interferon played a role in clearing normal microflora. While Type 1 interferons serve a protective purpose in some bacterial infections, in other cases, they exacerbate the infection, especially for secondary infections after a viral infection^38^. Our microbiome data reveal that patients with a high SARS-CoV-2 load have, (i) a distinct metagenomic composition, (ii) significantly reduced overall diversity (particularly decreased microbial diversity), and (iii) enhanced microbial dysbiosis signified by decreased F/B and loss of beneficial bacteria belonging to the *Firmicutes* and *Actinobacteria* families.

Overall, our study shows a strong correlation between interferon responses and viral load, which inversely correlates with clinical severity. We postulate an interplay of these three factors where a strong up-regulation of antiviral responses mediated by high viral load at the initial phase leads to protective immune responses. This postulation is supported by evidence in the literature that interferon responses are a protective factor for COVID-19^6,7,9,13,39^. Even though the size of the cohort was small, and the nature of the study was correlation-based, we show that a high viral load in the early symptomatic phase may contribute to a strong antiviral response in the host, which could lead to a mild outcome. However, it is not clear if a lower viral load at early infection can be considered an indicator for severe symptoms, considering that the majority of our patients showed a lower viral load. A follow-up study with a larger cohort could confirm our findings; more precise, traceable longitudinal studies should investigate the viral characteristics that contribute to imbalanced innate immune responses, leading to severe outcomes. Our study also suggests that besides the kinetic changes in viral load over the course of infection, the relationship between symptoms and viral load may also play a role in the massive spread of the virus in the community, where asymptomatic or mildly symptomatic SARS-CoV-2–infected people can actively interact with others. We propose to closely monitor and make use of the Ct value of each sample so as to prioritize the allocation of resources and minimize behaviors associated with higher risks of spreading viruses.

## Methods and Materials

### qRT-PCR to detect SARS-CoV-2 RNA

NP swab samples were collected and transferred in collection tubes with virus transport medium to the test site within 24 ~ 48 h. A total of 50 µL of sample was subjected to RNA isolation with a magnetic beads-based RNA isolation method (Direct-zol-96 MagBead RNA, Zymo Research). Then, isolated RNA was eluted in 50 µL of nuclease-free water. The RNA was subjected to a real-time reverse transcriptase–polymerase chain reaction (rRT-PCR) with the three primer and probes (N1, N2, N3, and human RNaseP) developed by the US CDC^14,40^ and TaqPath 1-Step RT-qPCR Master Mix, CG (Thermofisher). QRT-PCR was performed with StepOne Plus (Thermo), CFX96 (BioRad), or QuantaStudio Pro7 with the FAM channel. The threshold level was adjusted to minimize the background and to fall within the PCR exponential phase. No significant difference between the instruments was found. For copy number quantitation, a copy number standard template (IDTDNA) was used with a fivefold serial dilution starting from 200,000 to 1600 copies/µL.

### Cell culture

Vero E6 (ATCC CRL-1586) was purchased from ATCC and maintained in DMEM with 10% FBS. Cells were passaged weekly.

### Titration of virus in swab samples with the TCID50 assay

An NP swab sample of 0.4 mL of NP was diluted in 1.2 mL DMEM with 10% FBS, 1X penicillin and streptomycin, then filtered through 0.4 µm filter. The filtrate from the clinical swab sample was serially diluted by tenfold in DMEM with 10% FBS. The diluted samples were added to Vero E6 cells grown a 12-well plate, and 4 wells were used for each dilution (10-, 100-, 1000-fold diluted sample). Four days later, CPEs in the cells were recorded as the sign of infection and TCID50 was calculated followed by the Reed and Muench method^41^.

### Virus isolation

The filtrate (~ 0.5 mL) was added to Vero E6 cells seeded in a T-25 flask one day prior to infection for one hour at 37 °C. Then cells were replenished with fresh media and incubated at 37 °C in an incubator with 5% CO_2_. After a full CPE was monitored (at 4 to 5 DPI), the cell supernatant was harvested, and cellular debris was removed by centrifugation at 3000 rpm.

### Virus titration

Vero E6 cells grown in 24-well plates were incubated with viral samples diluted in the cell culture media. Vero E6 cells were seeded in 24-well plates and incubated to be confluent for 24 h. After cell supernatant was removed, tenfold serially diluted virus samples were added to each well (167 µL/well) and incubated for 1 h at 37 °C in a CO_2_ incubator. Cells were washed with PBS (1 ml/well) once, overlaid with DMEM with 0.5% methlycelluose and 10% FBS, and further cultured for 5 days. Cells were fixed and plaques by CPE were visualized by counter-staining with crystal violet.

### De novo RNA sequencing

Total RNA was isolated from samples with a magnetic beads-based RNA isolation method (Direct-zol-96 MagBead RNA, Zymo Research). A total of 200 µL of sample was used and then RNA was eluted in 25 µL. Libraries were prepared using the Ovation SoLo RNA-Seq Systems (NuGEN) with approximately10 ng of RNA. Libraries were subjected to a depletion of human and yeast rRNA using AnyDeplete Probe Mix (NuGEN) and (NuGEN). A total 1.3 mL of the final library at 1.8 pM, with 1% PhIX spike in was used for sequencing. Sequencing was performed on Illumina NextSeq 500 using the NextSeq 500/550 75 cycle High Output Kit v2.5 (20,024,906).

### Sequence pre-processing and assembly

The demultiplexed reads for each sample were assembled into preliminary contigs using MEGAHIT (v1.2.9)^42^. These preliminary contigs were then examined for their similarity to known Betacoronavirus sequences in order to merge them where possible, resulting in a preliminary viral assembly. The raw reads were then mapped back onto the preliminary assemblies using STAR (v2.6)^43^. Each position of the preliminary reference was further examined for locations varying from the actual reads using bcftools mpileup (v1.8)^44^, resulting in a small number of corrections to the preliminary reference. The raw reads were deposited into NCBI’s SRA (bioproject PRJNA626685) and the assembled viral genomes were deposited into GenBank (accessions MZ472095—MZ472108).

### SNP Analysis

Lastz^45^ was used to perform a pairwise alignment comparing the SARS-CoV-2 reference assembly NC_045512.2 against viral sequences of the isolates to identify locations having gaps, SNPs, and insertions/deletions across each pair.

### Metagenomics analysis

The sequences were aligned to the human hg38.p12 and SARS-CoV-2 reference NC_045512 reference genomes using STAR (version 2.6)^43^. Sequences not mapping to the host human genome were then analyzed for metagenomics composition using metaphlan2^46^. Functional profiling of metagenomics composition was performed using LEfSe^47^ to determine a significant linear discriminant analysis (LDA) score between groups.

### Ethic statement

The research has been reviewed and approved as Non Human Subjects Research and exempt from informed consent by Institutional Review Board of University of Louisville (IRB 20.0257 and IRB 05.0556). The study used residual specimens from clinical care de-identified by indirect identifier. Consent was exempted for these samples. Experiments were approved by Institutional Biosafety Committee of University of Louisville and were performed in accordance with relevant guidelines and regulations by Institutional Biosafety Committee of University of Louisville and US Centers for Disease Control and Prevension (Intrim guidelines for collecting, handling, and testing clinical specimens for COVID-19).

## Supplementary Information

Supplementary Information.
