# Supplementary material for: Induction of interferon response by high viral loads at early stage infection may protect against severe outcomes in COVID-19 patients
Source: Sci Rep. 2021 Aug 3;11:15715. doi: 10.1038/s41598-021-95197-y (PMC8333042; doi:10.1038/s41598-021-95197-y)
Supplement: Supplementary file 1 — Supplementary Information. [file 41598_2021_95197_MOESM1_ESM.docx]

## Supplementary Information

## Supplementary Table 1: Correlation analysis between various SARS-CoV-2 assays used in the study

| **Targets** | **CDC_N1Ct** | **CDC_N2Ct** | **LDT_N1** | **LDT_N3** | **IVD_N** | **IVD_orf** |
| --- | --- | --- | --- | --- | --- | --- |
| **CDC_N1Ct** | 1.000 | 0.995 | 0.958 | 0.957 | 0.958 | 0.893 |
| **CDC_N2Ct** |  | 1.000 | 0.969 | 0.968 | 0.970 | 0.914 |
| **LDT_N1** |  |  | 1.000 | 0.999 | 0.997 | 0.911 |
| **LDT_N3** |  |  |  | 1.000 | 0.997 | 0.906 |
| **IVD_N** |  |  |  |  | 1.000 | 0.921 |
| **IVD_orf** |  |  |  |  |  | 1.000 |

## Supplementary Table 2: Comparison of Ct values of the study samples from three independent SARS-CoV-2 assays

| **Samples** | **Ct values for** | | | | | |
| --- | --- | --- | --- | --- | --- | --- |
|  | **CDC** | | **LDT *** | | **IVD *** | |
|  | **N1** | **N2** | **N1** | **N3** | **N** | **orf** |
| KY-9A06 | 18.09 | 17.38 | 17.5 | 16.6 | NT | NT |
| KY-9A10 | 13.3 | 11.58 | 14 | 12.9 | NT | NT |
| KY-28D08 | 14.34 | 14.84 | 15.7 | 14.1 | 17.1 | 15.3 |
| KY-28F11 | 15.66 | 15.4 | 16.4 | 15.3 | 19.1 | 17.3 |
| KY-29G02 | 15.38 | 15.77 | NT | NT | NT | NT |
| KY-50G10 | 14.08 | 12.87 | 18.1 | 16.6 | 19.9 | 18.3 |
| KY-52D08 | 14.53 | 13.01 | 18.6 | 17 | 19.3 | 17.6 |
| KY-63G09 | 15.18 | 13.21 | NT | NT | NT | NT |
| KY-37H09 | 18.52 | 19.05 | 20.5 | 19.4 | 21.6 | 19.9 |
| KY-39C07 | 18.53 | 18.39 | 20.1 | 18.8 | 21.3 | 19.4 |
| KY-48H07 | 19.91 | 19.96 | NT | NT | 24.3 | 22.7 |
| KY-51C11 | 17.76 | 17.41 | 19.2 | 18.4 | 22.1 | 20.4 |
| KY-03G06 | 18.48 | 17.57 | 20.9 | 19.7 | 22.9 | 21 |
| KY-48D04 | 21.13 | 21.93 | 24.1 | 22.9 | 24.4 | 22.6 |
| KY-48E10 | 20.02 | 20.45 | NT | NT | NT | NT |
| KY-51C03 | 22.97 | 23.43 | 23.5 | 22.9 | 25.2 | 23.2 |
| KY-52F02 | 21.85 | 20.96 | 26.1 | 24.9 | 25.9 | 23.7 |
| KY-53C10 | 20.89 | 20.27 | 23 | 21.7 | 23.3 | 21.3 |
| KY-9E09 | 25.59 | 25.3 | 25.5 | 24.2 | NT | NT |
| KY-39C10 | 28.36 | 29.49 | 29.5 | 28.3 | 31.5 | 45 |
| KY-50F07 | 25.89 | 25.46 | NT | NT | NT | NT |
| KY-51H11 | 26.78 | 26.59 | 29.5 | 28.5 | 29.7 | 26.6 |
| KY-52D06 | 27.33 | 27.97 | NT | NT | NT | NT |
| KY-53B02 | 28.65 | 30.01 | 31.3 | 29.6 | 32.2 | 45 |
| KY-37A05 | 33.55 | 35.65 | 37 | 36.9 | 36.5 | 45 |
| KY-37D02 | 34.06 | 37.63 | Neg | Neg | Neg | Neg |
| KY-37F04 | 29.43 | 30.93 | 31.4 | 30 | 32.2 | 28.4 |
| KY-39H02 | 33.89 | 35.82 | Neg | Neg | Neg | Neg |
| KY-51H03 | 35 | 37.54 | NT | NT | NT | NT |
| KY-37A01 | Neg | Neg | Neg | Neg | Neg | Neg |
| KY-37A03 | Neg | Neg | Neg | Neg | Neg | Neg |
| KY-37A07 | Neg | Neg | NT | NT | NT | NT |
| KY-37A09 | Neg | Neg | NT | NT | NT | NT |
| KY-37B01 | Neg | Neg | NT | NT | NT | NT |

Samples for LDT and IVD analysis were 2-fold diluted in media (DMEM with 2% FBS)

NT : Not tested

Neg : Negative. For CDC and IVD assays, Ct > 39 were considered negative samples. For the LDT assays cut-offs of 38 and 37 were used for N1 and N3, respectively.

## Supplementary Table 3: Top 20 down-regulated genes for Top50% groups compared to the NegCtrl groups

| Ensembl ID | Gene Symbol\|Description | Log_2_FC | P-value | Q-value |
| --- | --- | --- | --- | --- |
| ENSG00000130176 | CNN1\|calponin 1 | -4.750 | 3.904e-11 | 9.102e-07 |
| ENSG00000140332 | TLE3\|transducin like enhancer of split 3 | -1.193 | 2.399e-07 | 0.001 |
| ENSG00000236969 | GGT8P\|gamma-glutamyltransferase 8 pseudogene | -3.810 | 1.566e-06 | 0.003 |
| ENSG00000164778 | EN2\|engrailed homeobox 2 | -2.838 | 6.194e-06 | 0.009 |
| ENSG00000142910 | TINAGL1\|tubulointerstitial nephritis antigen like 1 | -2.163 | 7.893e-06 | 0.009 |
| ENSG00000167751 | KLK2\|kallikrein related peptidase 2 | -2.504 | 1.174e-05 | 0.013 |
| ENSG00000108387 | SEPT4\|septin 4 | -2.491 | 1.323e-05 | 0.014 |
| ENSG00000167748 | KLK1\|kallikrein 1 | -2.830 | 2.042e-05 | 0.020 |
| ENSG00000124493 | GRM4\|glutamate metabotropic receptor 4 | -2.573 | 2.078e-05 | 0.020 |
| ENSG00000226979 | LTA\|lymphotoxin alpha | -3.045 | 2.690e-05 | 0.024 |
| ENSG00000132879 | FBXO44\|F-box protein 44 | -1.623 | 3.512e-05 | 0.027 |
| ENSG00000140479 | PCSK6\|proprotein convertase subtilisin/kexin type 6 | -2.032 | 4.393e-05 | 0.029 |
| ENSG00000160951 | PTGER1\|prostaglandin E receptor 1 | -3.183 | 5.042e-05 | 0.030 |
| ENSG00000105419 | MEIS3\|Meis homeobox 3 | -2.408 | 5.768e-05 | 0.032 |
| ENSG00000211895 | IGHA1\|immunoglobulin heavy constant alpha 1 | -3.016 | 5.877e-05 | 0.032 |
| ENSG00000143297 | FCRL5\|Fc receptor like 5 | -2.455 | 5.903e-05 | 0.032 |
| ENSG00000012124 | CD22\|CD22 molecule | -2.665 | 6.155e-05 | 0.032 |
| ENSG00000116337 | AMPD2\|adenosine monophosphate deaminase 2 | -1.407 | 7.689e-05 | 0.038 |
| ENSG00000224373 | IGHV4-59\|immunoglobulin heavy variable 4-59 | -3.121 | 8.641e-05 | 0.041 |
| ENSG00000124772 | CPNE5\|copine 5 | -2.270 | 8.732e-05 | 0.041 |

## Supplementary Table 4: Top 20 Enriched Gene Ontology biological processes identified using Cluster Profiler.

| GO ID | Description | P Value | Q Value | Count |
| --- | --- | --- | --- | --- |
| GO:0007611 | learning or memory | 1.25E-08 | 5.04E-05 | 92 |
| GO:0023061 | signal release | 1.80E-08 | 5.04E-05 | 143 |
| GO:0048167 | regulation of synaptic plasticity | 3.07E-08 | 5.04E-05 | 68 |
| GO:0060337 | type I interferon signaling pathway | 4.83E-08 | 5.04E-05 | 43 |
| GO:0071357 | cellular response to type I interferon | 4.83E-08 | 5.04E-05 | 43 |
| GO:0042391 | regulation of membrane potential | 6.62E-08 | 5.04E-05 | 125 |
| GO:0034340 | response to type I interferon | 7.10E-08 | 5.04E-05 | 44 |
| GO:0050804 | modulation of chemical synaptic transmission | 7.16E-08 | 5.04E-05 | 130 |
| GO:0099177 | regulation of trans-synaptic signaling | 8.44E-08 | 5.28E-05 | 130 |
| GO:0007215 | glutamate receptor signaling pathway | 1.08E-07 | 6.09E-05 | 41 |
| GO:0050890 | cognition | 1.20E-07 | 6.13E-05 | 100 |
| GO:0006836 | neurotransmitter transport | 1.58E-07 | 7.44E-05 | 87 |
| GO:1903305 | regulation of regulated secretory pathway | 1.97E-07 | 8.56E-05 | 56 |
| GO:0034765 | regulation of ion transmembrane transport | 2.24E-07 | 9.03E-05 | 144 |
| GO:0099643 | signal release from synapse | 2.48E-07 | 9.32E-05 | 60 |
| GO:0007269 | neurotransmitter secretion | 4.67E-07 | 0.000 | 59 |
| GO:0034341 | response to interferon-gamma | 5.05E-07 | 0.000 | 66 |
| GO:1902803 | regulation of synaptic vesicle transport | 5.42E-07 | 0.000 | 36 |
| GO:0045069 | regulation of viral genome replication | 6.76E-07 | 0.000 | 42 |
| GO:2000300 | regulation of synaptic vesicle exocytosis | 8.09E-07 | 0.000 | 34 |

Supplementary Table 5: Kruskal Wallis analysis of the microbial composition at the phylum level in four patient groups. Bayesian Q-value and FDR values: *<0.05, Trending= (0.051-0.07) and NS – not significant

| Phylum | Mild vs. NegCtrl | | Mild vs. Severe | | Moderate vs. NegCtrl | | Severe vs. NegCtrl | | Mild vs. Moderate | | Moderate vs. Severe | |
| --- | --- | --- | --- | --- | --- | --- | --- | --- | --- | --- | --- | --- |
|  | Q-value | FDR | Q-value | FDR | Q-value | FDR | Q-value | FDR | Q-value | FDR | Q-value | FDR |
| *Actinobacteria* | * | NS | * | Trending | NS | NS | NS | NS | NS | NS | NS | NS |
| *Viruses_noname* | * | * | * | Trending | NS | NS | NS | NS | NS | NS | NS | NS |
| *Pisuviricota* | * | NS | * | Trending | NS | NS | NS | NS | NS | NS | NS | NS |
| *Bacteroidetes* | * | NS | Trending | NS | NS | NS | NS | NS | NS | NS | NS | NS |
| *Synergistetes* | * | NS | NS | NS | NS | NS | NS | NS | NS | NS | NS | NS |
| *Proteobacteria* | * | Trending | NS | NS | NS | NS | NS | NS | NS | NS | NS | NS |
| *Firmicutes* | * | * | Trending | NS | NS | NS | NS | NS | NS | NS | NS | NS |
| *Ascomycota* | NS | NS | * | Trending | NS | NS | NS | NS | NS | NS | NS | NS |
| *Spirochaetes* | NS | NS | NS | NS | NS | NS | NS | NS | NS | NS | NS | NS |
| *Candidatus_*  *Saccharibacteria* | NS | NS | * | Trending | NS | NS | NS | NS | NS | NS | NS | NS |
| *Fusobacteria* | NS | NS | * | Trending | NS | NS | NS | NS | NS | NS | NS | NS |

Supplementary Table 6: Kruskal Wallis analysis for microbial families in four patient groups. Bayesian Q-value and FDR values: *<0.05, Trending= (0.051-0.07) and NS – not significant

| **Families** | **Mild vs. NegCtrl** | | **Mild vs. Moderate** | | **Mild vs. Severe** | | **Moderate vs. NegCtrl** | | **Severe vs. NegCtrl** | | **Moderate vs. Severe** | |
| --- | --- | --- | --- | --- | --- | --- | --- | --- | --- | --- | --- | --- |
|  | **Q-value** | **FDR** | **Q-value** | **FDR** | **Q-value** | **FDR** | **Q-value** | **FDR** | **Q-value** | **FDR** | **Q-value** | **FDR** |
| *Pseudomonadaceae* | * | Trending | NA | NA | NA | NA | NA | NA | NA | NA | NA | NA |
| *Bacillales_noname* | * | NA | NA | NA | NA | NA | NA | NA | NA | NA | NA | NA |
| *Lactobacillaceae* | * | Trending | NA | NA | NA | NA | NA | NA | NA | NA | NA | NA |
| *Coronaviridae* | * | NA | NA | NA | NA | NA | NA | NA | NA | NA | NA | NA |
| *Bifidobacteriaceae* | * | NA | NA | NA | NA | NA | NA | NA | NA | NA | NA | NA |
| *Micrococcaceae* | * | NA | NA | NA | Trending | NA | NA | NA | NA | NA | NA | NA |
| *Thermoanaerobacterales*  *_f_III_Incertae_Sedis* | * | Trending | NA | NA | NA | NA | NA | NA | NA | NA | NA | NA |
| *Gallionellaceae* | * | NA | NA | NA | NA | NA | NA | NA | NA | NA | NA | NA |
| *Aerococcaceae* | * | NA | NA | NA | NA | NA | NA | NA | NA | NA | NA | NA |
| *Alphaflexiviridae* | * | NA | NA | NA | NA | NA | NA | NA | NA | NA | NA | NA |
| *Paramyxoviridae* | * | NA | NA | NA | NA | NA | NA | NA | NA | NA | NA | NA |
| *Myoviridae* | * | NA | NA | NA | NA | NA | NA | NA | NA | NA | NA | NA |
| *Togaviridae* | * | Trending | NA | NA | NA | NA | NA | NA | NA | NA | NA | NA |
| *Clostridiales_f_XI_Incertae*  *_Sedis* | * | NA | NA | NA | NA | NA | NA | NA | NA | NA | NA | NA |
| *Streptococcaceae* | * | Trending | NA | NA | NA | NA | NA | NA | NA | NA | NA | NA |
| *Carnobacteriaceae* | * | NA | NA | NA | NA | NA | NA | NA | NA | NA | NA | NA |
| *Lachnospiraceae* | * | NA | NA | NA | NA | NA | NA | NA | NA | NA | NA | NA |
| *Veillonellaceae* | * | NA | NA | NA | Trending | NA | NA | NA | NA | NA | NA | NA |
| *Peptostreptococcaceae* | NS | NA | NA | NA | * | * | NA | NA | NA | NA | NA | NA |
| *Dermatophilaceae* | NS | NA | NA | NA | Trending | NA | NA | NA | NA | NA | NA | NA |
| *Saccharomycetales_noname* | NS | NA | NA | NA | Trending | NA | NA | NA | NA | NA | NA | NA |
| *Actinomycetaceae* | NS | NA | NA | NA | Trending | NA | NA | NA | NA | NA | NA | NA |
| *Retroviridae* | NS | NA | NA | NA | NA | NA | NA | NA | NA | NA | NA | NA |
| *Microbacteriaceae* | NS | NA | NA | NA | NA | NA | NA | NA | NA | NA | NA | NA |
| *Spirochaetaceae* | NS | NA | NA | NA | NA | NA | NA | NA | NA | NA | NA | NA |
| *Cardiobacteriaceae* | NS | NA | NA | NA | NA | NA | NA | NA | NA | NA | NA | NA |
| *Bacteroidetes_noname* | NS | NA | NA | NA | NA | NA | NA | NA | NA | NA | NA | NA |
| *Propionibacteriaceae* | NS | NA | NA | NA | NA | NA | NA | NA | NA | NA | NA | NA |
| *Potyviridae* | NS | NA | NA | NA | NA | NA | NA | NA | NA | NA | NA | NA |
| *Moraxellaceae* | NS | NA | NA | NA | NA | NA | NA | NA | NA | NA | NA | NA |
| *Eubacteriaceae* | NS | NA | NA | NA | NA | NA | NA | NA | NA | NA | NA | NA |
| *Synergistaceae* | NS | NA | NA | NA | NA | NA | NA | NA | NA | NA | NA | NA |
| *Porphyromonadaceae* | NS | NA | NA | NA | NA | NA | NA | NA | NA | NA | NA | NA |
| *Brevibacteriaceae* | NS | NA | NA | NA | Trending | NA | NA | NA | NA | NA | NA | NA |
| *Campylobacteraceae* | NS | NA | NA | NA | NA | NA | NA | NA | NA | NA | NA | NA |
| *Clostridiales_f_*  *XIII_Incertae_Sedis* | NS | NA | NA | NA | Trending | NA | NA | NA | NA | NA | NA | NA |
| *Candidatus_*  *Saccharibacteria_noname* | NS | NA | NA | NA | Trending | NA | NA | NA | NA | NA | NA | NA |
| *Neisseriaceae* | NS | NA | NA | NA | NA | NA | NA | NA | NA | NA | NA | NA |
| *Erysipelotrichaceae* | NS | NA | NA | NA | Trending | NA | NA | NA | NA | NA | NA | NA |
| *Enterobacteriaceae* | NS | NA | NA | NA | Trending | NA | NA | NA | NA | NA | NA | NA |
| *Bromoviridae* | NS | NA | NA | NA | NA | NA | NA | NA | NA | NA | NA | NA |
| *Corynebacteriaceae* | NS | NA | NA | NA | NA | NA | NA | NA | NA | NA | NA | NA |
| *Leptotrichiaceae* | NS | NA | NA | NA | NA | NA | NA | NA | NA | NA | NA | NA |
| *Flavobacteriaceae* | NS | NA | NA | NA | NA | NA | NA | NA | NA | NA | NA | NA |
| *Staphylococcaceae* | NS | NA | NA | NA | NA | NA | NA | NA | NA | NA | NA | NA |
| *Fusobacteriaceae* | NS | NA | NA | NA | NA | NA | NA | NA | NA | NA | NA | NA |
| *Pasteurellaceae* | NS | NA | NA | NA | NA | NA | NA | NA | NA | NA | NA | NA |
| *Enterococcaceae* | NS | NA | NA | NA | NA | NA | NA | NA | NA | NA | NA | NA |
| *Burkholderiaceae* | NS | NA | NA | NA | NA | NA | NA | NA | NA | NA | NA | NA |
| *Coriobacteriaceae* | Trending | NA | NA | NA | Trending | NA | NA | NA | NA | NA | NA | NA |
| *Prevotellaceae* | Trending | NA | NA | NA | NA | NA | NA | NA | NA | NA | NA | NA |

Supplementary Figure 1: PCoA plot for metagenomics beta diversity based on JACCARD index


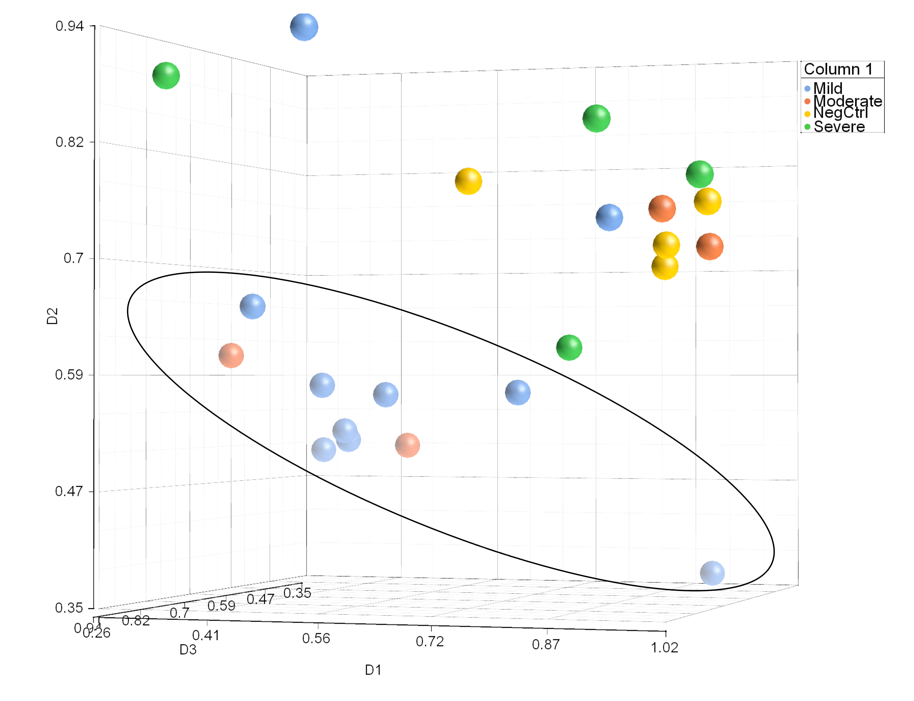


PCoA plot generated using matrix data with 3-dimensional scaling, Random Mapping, Method: Metric, Distance Function: Kendall dissimilarity and Max number of iterations: 100
